# Testing Replicability and Generalizability of the Time on Task Effect

**DOI:** 10.3390/jintelligence11050082

**Published:** 2023-04-28

**Authors:** Raimund J. Krämer, Marco Koch, Julie Levacher, Florian Schmitz

**Affiliations:** 1 Department of Psychology, University of Duisburg-Essen, Universitätsstraße 2, 45141 Essen, Germany; 2 Individual Differences & Psychodiagnostics, Saarland University, Campus A1.3, 66123 Saarbrücken, Germany

**Keywords:** time on task, response time, assessment, replication, figural matrices, conditional dependency, response processes

## Abstract

The time on task (ToT) effect describes the relationship of the time spent on a cognitive task and the probability of successful task completion. The effect has been shown to vary in size and direction across tests and even within tests, depending on the test taker and item characteristics. Specifically, investing more time has a positive effect on response accuracy for difficult items and low ability test-takers, but a negative effect for easy items and high ability test-takers. The present study sought to test the replicability of this result pattern of the ToT effect across samples independently drawn from the same populations of persons and items. Furthermore, its generalizability was tested in terms of differential correlations across ability tests. To this end, ToT effects were estimated for three different reasoning tests and one test measuring natural sciences knowledge in 10 comparable subsamples with a total *N* = 2640. Results for the subsamples were highly similar, demonstrating that ToT effects are estimated with sufficient reliability. Generally, faster answers tended to be more accurate, suggesting a relatively effortless processing style. However, with increasing item difficulty and decreasing person ability, the effect flipped to the opposite direction, i.e., higher accuracy with longer processing times. The within-task moderation of the ToT effect can be reconciled with an account on effortful processing or cognitive load. By contrast, the generalizability of the ToT effect across different tests was only moderate. Cross-test relations were stronger in relative terms if performance in the respective tasks was more strongly related. This suggests that individual differences in the ToT effect depend on test characteristics such as their reliabilities but also similarities and differences of their processing requirements.

## 1. Introduction

Since the very beginning of cognitive ability assessment, the evaluation of response time was a major concern in the psychology of individual differences (Spearman 1904). This is because time provides valuable information about the cognitive processes underlying a latent ability (De Boeck and Jeon 2019), over and above the accuracy of a response. Time on task (ToT) is the time from the presentation of an item to the registration of a response (Goldhammer et al. 2014). In recent years, ToT has received an increasing amount of attention, specifically in the case of complex cognitive tasks, such as reasoning tasks. This is first of all because computerized testing has become more popular in cognitive ability testing, and response times are readily available. Second, new and increasingly sophisticated approaches for modelling response time promise to provide novel insights and incremental information. These include diffusion models (Ratcliff 1978), various applications of the hierarchical model by Van der Linden (2007), branching models (Partchev and De Boeck 2012), models based on scoring rules for response time (Maris and van der Maas 2012) and models in the generalized linear mixed models (GLMM) framework (Goldhammer et al. 2014). A comprehensive overview of response time models is provided by De Boeck and Jeon (2019). However, a rapidly changing methodology and the use of insufficiently tested analysis techniques within a field also involve threats for the replicability of results. Replicability means the reliability of a previous result across different data (Nosek et al. 2022). Findings concerning the relationship of the processing time and the probability of successful task completion (i.e., the ToT effect; Goldhammer et al. 2014) vary in that they seem to depend strongly on participant and item characteristics. Consequently, the present study aimed to test the replicability of previously found result patterns and their consistency across comparable samples of persons and items, but also the generalizability of the ToT effect across different ability tests.

Epskamp (2019) discusses several problems concerning the replicability of empirical findings in the context of a fast-paced methodology. He stresses mistakes made during programming or analysis but also challenges with the implementation of new methods and their generalization to different tests and experimental paradigms. For example, there might be trouble in following the mathematical representations of the model or choosing the correct settings for model estimation. Replication is considered the ultimate criterion for the validity of empirical findings (Francis 2012). It ensures that knowledge can be separated from the context in which it was gained (i.e., a specific result pattern concerning the effects of response time on successful completion of a task is not limited to a particular sample), therefore it operationalizes objectivity (Schmidt 2009). However, the actual number of replications in psychological research has been shown to be alarmingly low in some domains (Makel et al. 2012). Furthermore, the results of systematic large-scale replication projects suggest that many published findings replicate with substantially lower effect sizes or not at all, possibly reflecting false positive results (e.g., Open Science Collaboration 2015; Klein et al. 2018). Consequently, a number of measures was proposed in order to increase replicability of empirical findings. This includes quantitative research synthesis and multiple replications (Maxwell et al. 2015). Although much has been achieved in this respect (Nosek et al. 2022), integration into day-to-day research is going slowly (Hardwicke et al. 2022). For the years 2014 to 2017, the prevalence of replication studies was still only at 5% (Hardwicke et al. 2022) and yet was substantially overestimated by the psychology research community (Agnoli et al. 2021).

### 1.1. Time on Task Effect

Traditionally, time on task effects have mostly been evaluated for relatively easy or elementary cognitive tasks (Kyllonen and Zu 2016). Parameters of the respective response time distributions have been shown to substantially relate to performance in complex cognitive tasks such as reasoning (Schmiedek et al. 2007; Schulz-Zhecheva et al. 2016). A joint consideration of response time and response accuracy within complex cognitive tasks has been growing in popularity since Van der Linden (2007) introduced the hierarchical model. The hierarchical model contains two separate measurement models, one for latent person ability and item difficulty, and one for latent person speed and item time intensity. These latent factors are again linked through higher order factors, representing the population of test takers or items. When applied to empirical data, the hierarchical model showed a dependency of ability and speed in complex cognitive tasks (Van der Linden 2007, 2009; Klein Entink et al. 2009; Goldhammer and Klein Entink 2011). However, there was no consistent pattern concerning the direction of the average effect, being positive for some and negative for other tasks. Based on findings that incorrect responses are generally associated with longer response times, one would expect solely positive dependencies of ability and speed. Importantly, this was shown for knowledge-independent (Hornke 2000) as well as knowledge-dependent (Lasry et al. 2013) tasks; therefore, was largely independent of the nature of the underlying cognitive process. Van der Linden (2009) suggested the wider range of correlations might be due to the better time management skills of able test-takers. This means, they slow down when there is enough time, resulting in a negative correlation, but also know how to efficiently distribute their time under time pressure, resulting in a positive correlation. Importantly, the hierarchical model assumes conditional independency of response time and response accuracy for a single item. The ToT effect would thus only reflect the relationship of latent person or item parameters (Van der Linden and Glas 2010). Conditional independency only holds when person ability and speed are constant across test items. Possible reasons why this might not always be the case are discussed by Bolsinova et al. (2017). Cognitive resources, for example, might decrease throughout a test due to fatigue. Speed might increase when a test-level deadline is approaching, at the cost of accuracy (the speed–accuracy trade-off; e.g., Wickelgren 1977). Finally, response processes might fundamentally differ across items, for example in the case of rapid guessing. A way to account for heterogenous response processes is the use of models with response classes. Using a branching model with separate branches for fast and slow responses, Partchev and De Boeck (2012) and DiTrapani et al. (2016) found different ToT effects for two self-paced cognitive tasks. For figural matrices, slower responses tended to be correct. By contrast, for verbal analogies, the ToT effects were small to nonexistent. It was concluded that fast and slow responses are based on different cognitive processes. In line with this, Molenaar et al. (2016) used a response mixture model, where every response by every subject was classified as either fast or slow. When applied to the Block Design subtest from the Wechsler Intelligence Scale in a sample of children it was shown that slow responses were less accurate. With a similar approach Molenaar and De Boeck (2018) explained the previous heterogenous results in terms of a moderation by task difficulty. For more complex tasks, slower responses had a higher probability of being correct.

This is in line with findings by Goldhammer et al. (2014) using a GLMM approach. By allowing model parameters to randomly vary across persons and items, conditional independency was not a necessary assumption. The ToT effect for a problem-solving task in that study was positive on average and increased further for situations of high demand; meanwhile, the ToT effect for a reading task was negative and decreased further for situations of low demand. Person ability and item difficulty jointly determine effective item demand, i.e., the resulting requirements in relative terms. This discrepancy between tasks was interpreted as evidence in support of two different processing modes. In line with the dual processing theory (Schneider and Shiffrin 1977) it was postulated that the direction of the ToT effect depends on whether the response process is automatic or controlled (Goldhammer et al. 2014). For easy tasks that can be solved largely automatically, such as reading, fast responses have a higher probability of being correct. By contrast, for difficult tasks that require controlled processes, slower responses have a higher probability of being correct. The assumption of different cognitive processes appeared plausible given that the TOT effect was shown to depend on task characteristics. However, in a subsequent study Goldhammer et al. (2015) found a negative effect of response time for a comparably difficult figural matrix task. Therefore, they adjusted theoretical considerations regarding the ToT effect in line with the mental model (Johnson-Laird 1994). Specifically, it was stated that at the final stage of an inductive process, the preliminary conclusion is evaluated taking into consideration possible inconsistencies between response options. If any inconsistencies are detected, the cognitive process is prolonged. If the inconsistencies are based on incomplete understanding a wrong response will be given, resulting in a negative ToT effect. However, Becker et al. (2016a) also found similar results for a matrix task with a construction response format that eliminates any inconsistencies between response options. The observed moderation effect of person ability and item difficulty (see also Goldhammer et al. 2014, 2015) was interpreted in line with cognitive load theory (Sweller 1994). Specifically, it was postulated that when the cognitive load of an item exceeds the cognitive resources of a person, controlled serial processing is required. By contrast, if cognitive resources exceed the cognitive load, automatic processes are assumed. This would account for the typical finding of a positive ToT effect for difficult items and a negative ToT effect for easy items, relative to person ability. Cognitive processes for items with moderate difficulty would therefore be predicted to be automatic for the more able and controlled for the less able persons.

The previously described modelling approach in the GLMM framework does not assume conditional independency, but also does not separate conditional dependencies from the effects of latent person or item characteristics. Bolsinova et al. (2017a) proposed a response moderation framework that allows modelling item and person differences in conditional dependencies. A model with item differences was applied to a low stakes knowledge test and resulted in an overall negative ToT effect, further decreasing for easy items (Bolsinova et al. 2017b). For a high-stakes arithmetic test, a model with item and person-by-item differences had the best fit. The overall ToT effect was positive, decreasing for easy items and able persons (Bolsinova et al. 2017a). Note that not raw but residual response times were used to determine the ToT effect. This means time on task reflected relatively fast or slow responses, given person speed and item time intensity. In no way does this refute theoretical explanations for the ToT effect in the sense of dual processing or cognitive load theory. On the contrary, it suggests that they apply with a certain variability on the level of single responses that cannot fully be explained by the underlying latent person and item variables.

To conclude, the ToT effect appears to be complex and there is only limited evidence that would allow to estimate its generalizability across different contexts. Naumann and Goldhammer (2017) compared the ToT effect for a digital reading task across samples from 19 different countries using PISA 2009 data. For this purpose, the average ToT effect for each country was submitted to a random effects meta-analysis. The meta-analytic effect was positive, but with significant heterogeneity across countries. In a second model, interaction terms of time on task with both person ability and task demands were included. Note that person ability and task demands were not operationalized by the relative number of correct responses in the digital reading task, as executed in previous research. Instead, person ability was determined by an independent print-reading task and task demands by the number of navigational steps necessary to complete the digital reading tasks. The meta-analytically estimated interaction terms of time on task with both person ability and task demands were significant and in the expected direction. Specifically, they indicated more positive ToT for weak participants solving difficult items, in relative terms. Only the moderation of person ability varied significantly across countries.

Using the same approach and data, Naumann (2019) showed that the time spent on a digital reading task did not only depend on item difficulty, but also on ability, enjoyment of reading and knowledge of reading strategies. The fact that person characteristics have a significant influence on time on task in self-paced tasks raises the question how the ToT effect changes when persons are forced to give a response within certain time constraints. This is not only interesting from a theoretical point of view but time constraints are frequently applied in assessment for reasons of efficiency. The adverse effects of time constraints on accuracy have been extensively studied in terms of the speed–accuracy trade-off. Importantly, under conditions of item- or test-level deadlines the number of correct responses do not reflect ability in the classical sense but rather the effective problem-solving rate (Kyllonen and Zu 2016). These are distinguishable constructs, as can be inferred by a lack of measurement invariance between conditions with and without time constraints (De Boeck et al. 2017) and timed ability only being moderately correlated with untimed ability (Wilhelm and Schulze 2002; Goldhammer et al. 2021). For a word recognition task, Goldhammer et al. (2017) found positive effects of residual time on task on response accuracy in four timed conditions and no significant effect in an untimed condition. However, it is unresolved to what extent this effect might have been affected by responses outside the response window that were also analyzed in the timed condition. De Boeck et al. (2017) found ToT effects to only slightly differ in a subject-paced as compared to an experimenter-paced version of a numerical reasoning test, with all of them being significantly negative.

Finally, Domingue et al. (2022) investigated the heterogeneity of the ToT effect across 29 datasets, featuring different cognitive tasks and dissimilar samples. The moderating effect of item difficulty and person ability varied widely across datasets, and, in general, response time was not a strong predictor of accuracy. This study provides an impressive overview of the ToT effect for different types of cognitive tasks. However, since the data stem from different tasks and different samples, the relative roles of related moderators remain unresolved. In fact, the observed heterogeneity could reflect two sources: (1) The ToT effect itself is not reliable and even close replication (i.e., with highly comparable samples, materials, and analysis methods) is not possible. In the alternative (2), the ToT effect strongly depends on context and is potentially moderated by relevant variables. The latter questions the generalizability of the ToT effect and suggests its extension is limited to boundary conditions.

It is not only the theoretical understanding of ToT effects that has increased in recent years. The consideration of response time for the improvement of testing practices and ability assessment has gained popularity as well (Margolis and Feinberg 2020). This includes the use of time on task as a predictor of rapid guessing behavior (e.g., Jin et al. 2022; Rios 2022), disengaged responses (Nagy and Ulitzsch 2022) or item pre-knowledge (Lee and Wollack 2020). In general, there is a development towards including more information than only the number of correct responses as an indicator of latent cognitive ability. Approaches such as the cognitive diagnostic model (e.g., Hsu et al. 2020; Zhan et al. 2021) propose the integration of response time and other variables such as eye-tracking data into a joint framework of ability assessment. There is also evidence suggesting that consideration of time on task can improve the precision (Reis Costa et al. 2021) as well as the efficiency (Kern and Choe 2021) of ability assessment.

### 1.2. Motivation and Goals

By considering response time, valuable information can be gained about the processes underlying cognitive abilities. Empirical findings consistently show a relationship of time on task and successful completion of the task. By using new methodologies to jointly model response time and response accuracy, understanding of the ToT effect has increased continuously. This includes the identification of the relationship as being moderated by item difficulty and person ability (Goldhammer et al. 2014, 2015; Becker et al. 2016a). The moderation effects can be reconciled with both dual processing and cognitive load theory. In the case of easy tasks or the high ability of the respondent, the response process is increasingly automatic and fast answers tend to be correct. In the case of difficult tasks or less able respondents, a controlled process is required, and slow answers tend to be correct. This theory is supported when considering local dependencies (Bolsinova et al. 2017a, 2017b) as well as for conditions with time constraints (De Boeck et al. 2017; Goldhammer et al. 2017). However, studies investigating the ToT effect are based on various task types and information is often derived from simulated data. Although application of different models to real data has been shown to result in converging results, homogeneity of the ToT effect across task types has rarely been quantified. There is no information available on whether person or task characteristics outweigh in terms of size and direction of the effect. Either possibility bears important implications for the assessment of cognitive abilities and the underlying cognitive processes. If the relationship of response time and response accuracy is stable and person specific, one might consider adjusting individual test scores for time on task (e.g., Reis Costa et al. 2021) or using scoring rules that incorporate response times (Maris and van der Maas 2012; van Rijn and Ali 2017, 2018).

Replication is especially being called for in fields with fast changing methodology and results. The goal of the present study is, in part, a close replication of the results reported by Goldhammer et al. (2015) and Becker et al. (2016a) and the evaluation of their consistency across subsamples in the current study. A close replication means recreating the methods of a previous study as closely as possible (Brandt et al. 2014). In part, it is also investigating the homogeneity of the ToT effect across different tasks, providing information about the generalizability of the ToT effect across different contexts. Further, by using correlational and meta-analytical methods, quantitative criteria for the consistency of the ToT effect are provided. Specifically, this study aims to investigate:

1. Replicability of the ToT effect with respect to size and direction of the effect across independent samples of persons drawn from the same population and items drawn from the same type of figural reasoning task;
2. Replicability of the moderation of the ToT effect by person ability and item difficulty across independent samples of persons drawn from the same population and items drawn from the same type of figural reasoning task;
3. Generalizability of the ToT effect across different types of complex cognitive tasks.

## 2. Materials and Methods

### 2.1. Data Acquisition and Sample

All participants were registered applicants of the admission tests for German medical schools in 2021. Data collection took place as part of an online training session for the pending admission tests. There were four subtests, three measuring figural, numerical and verbal reasoning and one subtest measuring natural sciences knowledge. It was an unsupervised and self-paced study without time limits. Participants were allowed to take breaks between but not within each subtest. Every item was presented on a single page and the survey registered the time in seconds until the send button was pressed. The four subtests were administered in a randomized order.

Of *N* = 4527 participants who completed the four subtests, 10 individuals older than 40 years were excluded. This is because reasoning ability has been shown to decrease over time due to diminishing speed of processing beyond the age of 20 (Salthouse 1996; Nettelbeck and Burns 2010). Therefore, ToT effects of individuals older than 40 years might not be comparable to the generally young sample. Further exclusion criteria were an average response time below six seconds (153 participants) or zero correct responses (76 participants) or both (1603 participants) for either one of the four tasks. Six seconds was estimated to be the minimum time required to read and understand item requirements. Participants with extremely long average response times (*n* = 45) were discarded following visual inspection of the response time distribution of the respective task. The remaining final sample size was *n* = 2640. The process of eliminating extreme responses differed from Goldhammer et al. (2015) and Becker et al. (2016a), both of which comprised elimination of logarithmized response times more extreme than *M* ± 3*SD*. This approach was not possible in the present study due to the high number of participants who responded extremely fast and inaccurately. This extremely skewed response-time distribution is presumably an artefact of the context in which the data collection took place. Many participants might have merely observed the items to become familiar with what they can expect in the admission test for which they were training. In the alternative, they may have just clicked through these tasks in order to see the other tests. However, we believe that after applying the exclusion criteria described above, the sample was mostly limited to test takers who seriously intended to solve the tasks. This is also suggested by the descriptive statistics (see Section 3.1) of the tasks that were all within a plausible range and comparable to what was reported by Goldhammer et al. (2015). Therefore, we believe that the conditions required for a replication of the desired result patterns of the ToT effect are given.

Due to a coding error, 560 participants had missing data in one item of the figural reasoning subtest. No mean scores were calculated for these participants, but they were nevertheless considered for the estimation of ToT effects. The age of the final sample ranged from 17 to 40 years with a mean of *M*(*SD*) = 21.34(2.48). Participants were 75% female and about half voluntarily reported their grade point average at the time of graduation from high school (*M*(*SD*) = 1.65(0.53), where grade one indexes the best performance in the German Abitur and grade four the worst possible grade).

### 2.2. Materials

**Figure 1:**
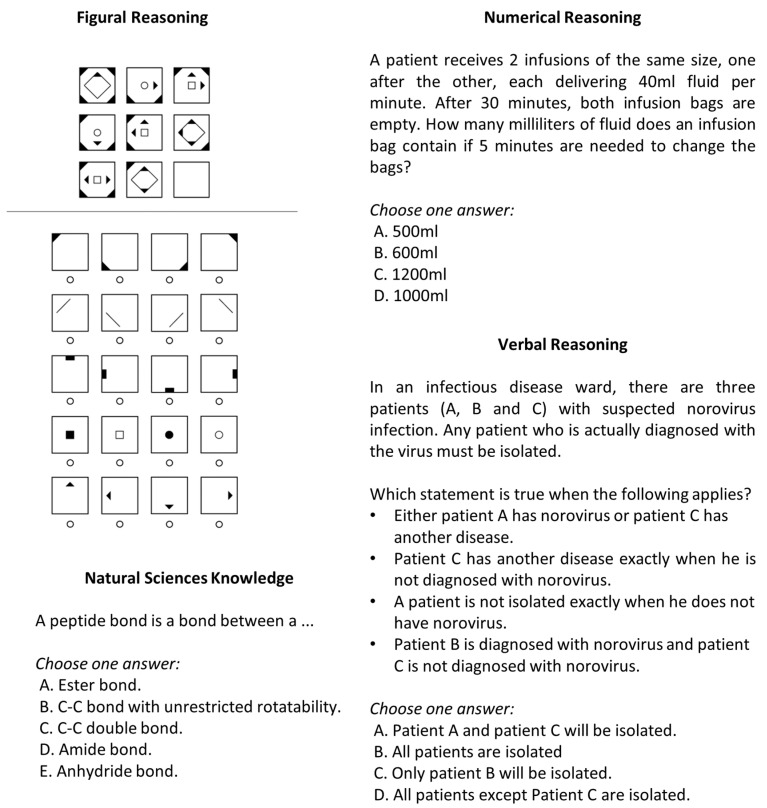
Example Items of Cognitive Ability Tests.

Example items of the four cognitive ability measures are provided in Figure 1. Figural reasoning was measured using a figural matrix task. For the present study, 10 different test sets of 28 items were constructed, including 6 linking items that were administered in each test set (for more detailed information, see Koch et al. 2022). Participants were randomly assigned to a test set during data collection. The matrices consisted of nine cells, eight of which were filled with geometric symbols arranged according to certain rules (e.g., addition, subtraction, completeness). Answers were given in a construction response format, meaning test takers had to fill the last empty cell by adding symbols which logically completed the matrix. The number of rules per item varied from one to five.

Numerical reasoning was measured through 16 logical arithmetic text problems that were based on a rule of three. Answers were given in a multiple-choice format with four options.

The verbal reasoning task comprised 16 items including text-based premises. Test takers had to compare and analyze the given relations to answer a corresponding question. The response format was identical to that of the numerical reasoning test. Items within each reasoning subtest were presented in ascending order of difficulty. Numerical and verbal reasoning tasks were contextualized for a medical context.

The natural sciences task included 20 questions in biology, chemistry, physics and mathematics at the level of high school graduation. Therefore, prior knowledge was necessary to solve them. All items in this subtest were presented in a multiple-choice format with five response options.

### 2.3. Data Treatment and Statistical Analyses

To investigate the replicability of the ToT effect we followed the approach of Goldhammer et al. (2014, 2015), and Becker et al. (2016a), that is based on a 1-Parameter Logistic (1PL) item response model in which not only person- but also item parameters are treated as random variables (random person random item model; De Boeck 2008). Technically, this analysis corresponds with a generalized linear mixed model (Baayen et al. 2008) in which the logit of the probability of a correct response ${ⴄ}_{pi}$ is regressed on log-transformed, grand-mean centered response time $t_{pi}$.

(1) $${ⴄ}_{pi}=\beta_{0}+b_{0p}+b_{0i}+\left(\beta_{1}+b_{1p}+b_{1i}\right)t_{pi}$$

The fixed intercept $\beta_{0}$ corresponds to the logit of the probability of a correct response across all items and persons. Adjusted by the random intercept across persons $b_{0p}$ or items $b_{0i}$, this value reflects person ability or item easiness. The fixed slope for response time $\beta_{1}$ corresponds to the overall ToT effect. This is the logit probability of successful task completion as determined by time investment. Adjusted by the random slope across persons $b_{1p}$ or items $b_{1i}$, time on task can be interpreted as a person-specific parameter varying by a task’s time intensity, or an item-specific parameter varying by person’s speed (Goldhammer et al. 2014).

All analyses were conducted using R 4.2.2 (R Core Team 2022). To fit several GLMM models as described by De Boeck et al. (2011), we used the package lme4 (Bates et al. 2015). A separate model was fitted for each task, i.e., for numerical reasoning, verbal reasoning and natural sciences knowledge. One model was fitted across all items and two models were fitted separately for the two test halves to obtain a measure of split-half reliability $\rho_{b1p}$ as uncorrected correlation of the $b_{1p}$ estimates. For this purpose, test items were sorted according to difficulty and then split alternately (ABAB). In case of figural reasoning, 30 models were fitted in the same manner but for each of the 10 subsamples separately. With the exception of the figural reasoning models, a guessing probability of 20% or 25% was implemented in the link function to account for guessing in the alterative forced-choice test with the five or four response options, respectively (Knoblauch 2014). Prior to parameter estimation, single responses given within the first five seconds after stimulus onset, and responses given after 250 s or more, were excluded from the respective models only. While five seconds were not considered a sufficient time to understand item requirements, it was assumed that responses slower than 250 s likely reflect a non-continuous (i.e., an interrupted) response process. Subsequently, response times were log-transformed and grand-mean centered. Note that raw response times were used, and not residuals as used by Bolsinova et al. (2017b). This is because for the purpose of this paper it is not necessary to separate the speed–ability correlation from the conditional dependence between response time and accuracy.

To investigate replicability of the ToT effect across figural reasoning subsamples, a meta-analytical approach was chosen. First, a random effects meta-analysis using the fixed slope estimates $\beta_{1}$ weighted by their reciprocal standard errors was conducted with the metafor package (Viechtbauer 2010). In a second step, conditional accuracy functions were predicted based on the general linear mixed models. Separate functions were predicted for each sample and three levels of person ability and item difficulty (low, mean, high). For this purpose, persons and items were split into equal thirds according to the respective mean of correct responses. To each combination of sample, ability and difficulty a general additive model (GAM) was fitted using the mcgv package (Wood 2011). The model-implied estimates were, then, meta-analyzed and visualized using the metagam package (Sørensen et al. 2021). Smooth functions were based on cubic spline interpolation with four knots.

To investigate generalizability in terms of the breadth of the ToT effect across different types of tasks, bivariate correlations of the $b_{1p}$ estimates were calculated. This was performed first for the total sample, then, separately for all subsamples. For this purpose, another 10 GLMMs each for numerical reasoning, verbal reasoning and natural sciences knowledge were fitted. Note that these models were based on identical items for each subsample, differently from figural reasoning where varying items were used.

Finally, a composite cognitive-ability score was computed as the mean of the respective task means from numerical reasoning, verbal reasoning and natural sciences knowledge. The composite score served the purpose of investigating the moderation of person ability with an ability measure that was not based on the same task as the ToT estimates, in order to reduce the risk of computational dependency. This composite score was, in turn, correlated with the figural reasoning $b_{1p}$ estimates.

## 3. Results

### 3.1. Descriptive Statistics

Descriptive statistics and bivariate correlations of the four cognitive performance measures are given in Table 1. All tasks were comparable in terms of empirical difficulty (ranging from *M*(*SD*) = .49(.18) for natural sciences knowledge to *M*(*SD*) = .64(.26) for figural reasoning) and time intensity (ranging from *M*(*SD*) = 54.44(14.46) for figural reasoning to *M*(*SD*) = 57.26(6.83) for numerical reasoning). However, internal consistency of the accuracy scores was only moderate in the case of numerical reasoning and natural sciences knowledge (ω = .68) and even lower in the case of verbal reasoning (ω = .55). Consequently, the uncorrected ability correlations were weak (*r*(2638) = .24, *p* < .001 for the correlation of verbal reasoning and natural sciences knowledge) to moderate (*r*(2078) = .40, *p* < .001 for the correlation of numerical reasoning and natural sciences knowledge). Internal consistency of response times was higher (.75 ≤ ω ≤ .81). The corresponding correlations were weak but comparable for the different tasks (.16 ≤ *r* ≤ .30).

### 3.2. Replicability of the Time on Task Effect for the Figural Reasoning Task across Samples

**Figure 2:**
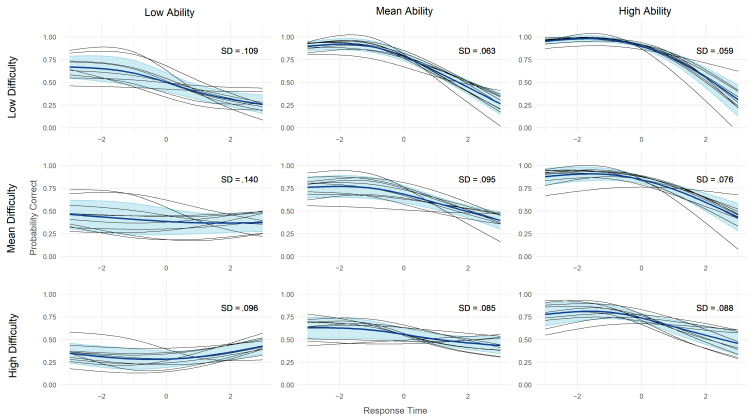
Probability of a correct answer in the figural reasoning task as a function of response time, split by item difficulty, person ability and subsample. Model implied conditional accuracy functions, averaged across persons within subsamples. Log-transformed response time is centered. SD = mean standard deviation across subsamples and all data points. Black lines represent subsamples; dark blue lines represent the meta-analytic mean, light blue areas represent one standard deviation above and below the mean.

The random effects meta-analysis of the 10 figural reasoning subsamples resulted in an overall ToT effect of $\beta_{1}$ = −0.60 (95% CI [−0.74, −0.47], *I*^2^ = 13%; see Figure S1 (Supplementary Materials)). Fixed slope estimates were all negative with no significant heterogeneity and ranged from $\beta_{1}$ = −1.21 (95% CI [−1.68, −0.74]) for sample 1 to $\beta_{1}$ = −0.22 (95% CI [−0.68, 0.25]) for sample 9. For all samples but sample 9 (*r* = −.06) there was a negative correlation of the $b_{0p}$ and $b_{1p}$ estimates (*r* = −.38 to *r* = −.72), meaning stronger negative ToT effects for more able persons. Similarly, the item correlations *r*($b_{0i}$,$b_{1i}$) were negative on average (*r* = −.24), meaning stronger negative ToT effects for easier items. However, the correlations were smaller and less consistent, with positive correlations for sample 2 (*r* = .34) and 9 (*r* = .14). Figure 2 shows the model implied and meta-analyzed conditional accuracy functions, split by item difficulty and person ability. Generally, as can be observed by the dark blue lines, faster answers were more likely to be correct. However, the size and direction of the relationship varied stronger as item difficulty increases and participant ability decreases. A positive overall ToT effect was even observed for participants with low abilities solving difficult tasks. Concerning replicability of the ToT effect, the black curves representing the different samples were largely comparable. Heterogeneity across samples was higher for low ability participants (*SD* = 0.096–0.140) as compared to mean and high ability participants (*SD* = 0.059–0.095).

### 3.3. Generalizability of the Time on Task Effect across Tasks

Model parameters and bivariate correlations of the $b_{1p}$ estimates for the four cognitive performance measures are given in Table 2. As compared to figural reasoning, the overall ToT effects ($\beta_{1}$) were less negative for numerical reasoning ($\beta_{1}$ = −0.23) and natural sciences knowledge ($\beta_{1}$ = −0.08) and even positive for verbal reasoning ($\beta_{1}$ = 0.34). The correlations of the $b_{0p}$ and $b_{1p}$ estimates were negative for all tasks (*r* = −.21 for verbal reasoning to *r* = −.60 for natural sciences knowledge). The correlations of item random effects *r*($b_{0i}$,$b_{1i}$) were once again more heterogenous, with a strong negative relation of item easiness with the ToT effect in the case of numerical reasoning (*r* = −.77), but virtually no relations for verbal reasoning and natural sciences knowledge. Split-half reliability was moderate for figural reasoning ($\rho_{b1p}$ = .41) and natural sciences knowledge ($\rho_{b1p}$ = .40), but low for numerical reasoning ($\rho_{b1p}$ = .22) and verbal reasoning ($\rho_{b1p}$ = .04). The stability of the ToT effect across task types, as reflected in the bivariate correlations of the respective random slopes across persons, was low. It ranged from r(2638) = .08, *p* < .001 for the correlations of verbal reasoning with figural reasoning and numerical reasoning to r(2638) = .22, *p* < .001 for the correlation of numerical reasoning and natural sciences knowledge.

**Figure 3:**
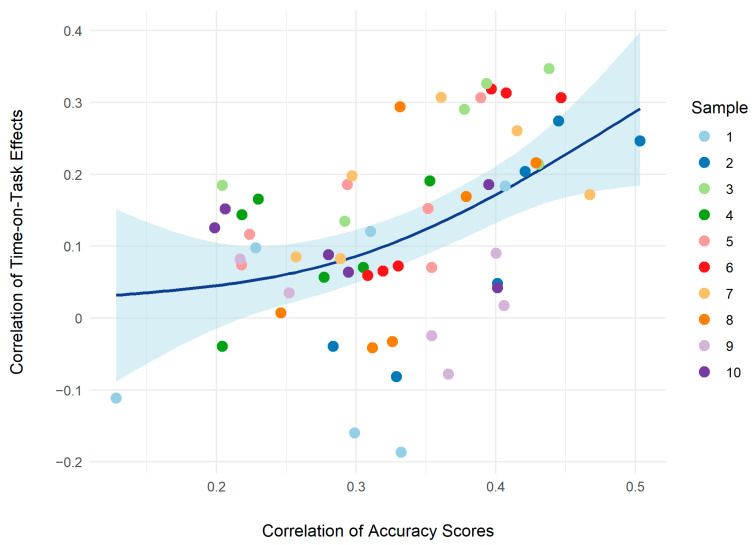
Bivariate correlations of the time on task effects as a function of bivariate ability correlations. Time on task estimates are based on separate models for each of the four cognitive ability measures times the 10 subsamples. The dark blue line represents the smoothed overall trend with its 95% confidence interval in light blue.

Figure 3 shows the bivariate correlations of the ToT effects as a function of the respective bivariate ability correlations for the 10 samples. The dark blue line reflects the overall trend, in that the ToT correlations increased curvilinearly with increasing correlations of the abilities they were based on. Finally, the composite score of numerical reasoning, verbal reasoning, and natural sciences knowledge was moderately correlated with figural reasoning accuracy (*r*(2078) = .42, *p* < .001). The correlation of the composite score with the figural reasoning ToT effect was *r*(2638) = −.22, *p* < .001. This means that for participants with higher general cognitive ability, faster answers tended to be correct.

## 4. Discussion

In the present study, we investigated the replicability and generalizability of the ToT effect within and across four different cognitive tests. The overall ToT effect for the figural reasoning test was negative, meaning fast responses tended to be correct on average. Negative ToT effects have indeed been reported for studies utilizing the same task type (e.g., Goldhammer et al. 2015; Becker et al. 2016a). In terms of dual processing theory, this suggests relatively non-effortful (“automatic”) processing, which seems to contradict classical understanding of reasoning. According to Klauer et al. (2002), matrices demand detection of similarities, as well as differences between objects and therefore represent one of the most complex inductive problems. However, the effective mental load generated by an item depends on the ability of the test taker and the number of item rules and their interactivity. In the present test, the number of rules varied from one to five, which is quite typical. Nevertheless, the test was the easiest as compared to the other three cognitive tests. Furthermore, the ability level in the present sample can be presumed to be high. Test takers were applicants of the admission tests for German medical schools and reported to have excellent school grades in line with admission requirements. Finally, while the number of rules increased across items, the type of rules remained the same. Thus, able participants might have inferred the rules from the early items and applied this knowledge to later items, thereby, processing them in a less effortful fashion. Rule knowledge has been shown to increase response accuracy in figural matrix tasks (Levacher et al. 2022).

The moderation effect of item difficulty and person ability within the figural reasoning task replicated the pattern reported by Goldhammer et al. (2015) and Becker et al. (2016a). Specifically, as items became more demanding (i.e., with decreasing ability and increasing item difficulty), the accuracy of responses increased with processing time. In line with these findings, we observed a positive ToT effect for persons with low ability solving difficult items. This suggests that only for this condition the cognitive load of the items on average exceeded the cognitive resources of the test takers, requiring mostly controlled processing in order to give a correct response. This specific pattern replicated well across different samples of items and persons, although the correlations of item random effects were more heterogenous. Given that all subsamples of persons were randomly drawn from the same population of applicants, and items were generated in an analogous fashion, comparable person abilities and item difficulties were to be expected.

The mean accuracies for the figural reasoning subsamples ranged from .50 for sample nine to .72 for sample one. Accordingly, these were the samples with the least and the most negative ToT effects, respectively. Heterogeneity across samples was highest for low ability test-takers. This is plausible as well, since low ability test-takers seem to also be more variable in their responses (Ratcliff et al. 2008; Schmiedek et al. 2007). Heterogeneity between samples was especially high for low ability persons solving items of mean difficulty. This is because for some samples the ToT effect was negative and for others positive. It therefore represents the point where cognitive load and cognitive resources were in balance, and where a relatively minor variation could determine the direction of the ToT effect. Another possible explanation for differences in heterogeneity are lower goal-management skills in persons with lower working memory capacity (Carpenter et al. 1990; Krieger et al. 2019), resulting in the use of different, and potentially more error-prone, strategies (Li et al. 2022). At the least, the construction response format prevented response elimination strategies as a means to (differentially) simplify the solution process (Becker et al. 2016b).

Finally, generalizability of the ToT effect across different types of tasks was low. The overall ToT effects for the four cognitive tests differed in size as well as in direction. Surprisingly, the effect for the verbal reasoning task was positive, therefore slower responses had a higher chance of being correct. This cannot be explained by the higher difficulty of the items since the mean accuracy score was basically the same as for the figural reasoning task. However, the internal consistency of the verbal reasoning task was fairly low for an ability measure with 16 items, and the ToT effects for two test halves did not correlate meaningfully. This suggests that there was a high heterogeneity of response processes across items within that specific task type.

Low reliability may be one factor that reduces the correlation of ToT effects between tasks. Another factor is the actual differences in the required cognitive processes and required competencies. In the case of verbal- and figural reasoning these differences are presumably low. Both abilities require creation and manipulation of mental representations and therefore fundamentally rely on working memory capacity (Wilhelm 2004). Empirical findings show that the performance in reasoning tasks can be explained by working memory capacity to a large extent, independent of task content (Kyllonen and Christal 1990; Süß et al. 2002). The same line of argumentation applies to quantitative reasoning. The correlation of ToT effects between test halves was low and presumably outweighing generally different response processes as a possible explanation for low inter-task correlations. In this respect, natural sciences knowledge stated the only exception. Reliability of the ToT effects was considerably higher as compared to the verbal and numerical reasoning, but the tasks arguably tap different abilities. The three reasoning tasks are indicators of fluid intelligence, whereas natural sciences knowledge is an indicator of crystallized intelligence (Cattell 1963). The different cognitive processes involved might likely account for the low correlation of ToT effects between natural sciences knowledge and the reasoning tasks.

The moderation of the ToT effect by person ability was comparable across tasks, whereas the moderation by item easiness varied more widely. The expected negative correlation of item random effects could be observed for numerical reasoning and to a lesser extent for figural reasoning, in line with previous findings by Goldhammer et al. (2014) for reading literacy and problem solving. Conversely, there was no substantial correlation for verbal reasoning and natural sciences knowledge in the present study. The latter finding suggests that knowledge represents a special case, as empirical difficulty is largely independent of processing requirements, but primarily depends on whether a piece of information has been previously learned or not. The absence of a negative relation for verbal reasoning was not expected. It may reflect the previously discussed heterogenous response processes that may be additionally moderated by specific item requirements unrelated to difficulty.

In summary, item difficulty and person ability could account for differences in the direction and size of the ToT effect for the figural matrix task. Therefore, the accounts on dual processing and cognitive load may provide an explanation for within-task differences of the ToT effect. By contrast, differences of ToT effects between tasks could be better accounted for in terms of qualitatively different processes. However, this does not refer to the degree of automation, since the cognitive tests were mostly comparable in terms of difficulty and, more importantly, the overall ToT effects.

Cross-task correlations of the ToT effect seem to be related with the abilities assessed by the respective tests. This can be inferred from the finding that the correlations of ToT effects increased with increasing correlations of the test performances. Note that this relation is likely estimated at its lower bound, given that ability correlations were restricted in the current sample, with only one ability correlation exceeding *r* = .50. To address challenges associated with compromised reliability of the single test scores, we computed a composite score of mental ability based on numerical reasoning, verbal reasoning and natural sciences knowledge. The correlation of the composite score with the figural reasoning ToT effect was negative, meaning that for participants with higher general cognitive ability, faster answers tended to be correct. This is in line with accounts on dual processing and cognitive load, suggesting that more capable persons solve items in a less effortful fashion. However, the fact that only a small portion of variance in the figural reasoning ToT effects could be accounted for calls into question the extent to which these theories can be applied to between-task differences.

### Limitations

In spite of finding plausible relations that generally replicated core findings in the literature, variance restrictions were likely in the current sample. All samples comprised relatively strong students with a limited range of excellent graduation results. At the same time, this study took place in a low-stakes setting that served as voluntary training only. This was reflected in the large number of test takers who had to be excluded due to fast response times. It can be assumed that a considerable proportion of those remaining in the sample also did not exert maximum effort. Furthermore, in some cases, test takers might have interrupted or prolonged the response process to familiarize themselves with the respective task type. Therefore, we excluded participants with extremely long response times. The results of this study require further consolidation in a high-stakes setting using a more heterogenous sample. Additionally, the effects of reduced effort and other non-cognitive factors on size and direction of the ToT effect should be considered. There were some limitations also concerning the reliability of the measures. In part, this may be attributed to the setting of the study. Another limitation concerns the approach that was chosen to model the effects of time on task on response accuracy. Generalized linear mixed models with random effects across persons as well as items are highly complex. Specifically, the number of items between 16 and 28 was very low for this analytical approach (e.g., McNeish and Stapleton 2016). This issued convergence warnings and low standard errors for some of the models. To ensure that the ToT estimates were unaffected by these problems, models were fit with various estimators and optimizers (R Package Documentation 2022), all leading to virtually identical estimates. However, the item estimates $b_{1i}$ are potentially unreliable and should only be interpreted with caution.

Another point that deserves discussion is that only linear terms were modelled. Naumann and Goldhammer (2017), for instance, found evidence for quadratic ToT effects in a digital reading task. Furthermore, there are several recent findings suggesting that conditional dependencies of response accuracy on response time follow a curvilinear inverted U-shaped function (e.g., Chen et al. 2018; Bolsinova and Molenaar 2018; Kang et al. 2022). In view of this, we reanalyzed the figural reasoning data with models comprising a fixed quadratic effect of response time. Inclusion of this term did improve model fit, but the increase in explained variance was not meaningful. Modelling curvilinear dependencies or quadratic ToT effects with variation across items and persons would require even larger samples or Bayesian methods with strong priors.

## 5. Conclusions

The present study aimed to investigate the replicability of the ToT effect and its generalizability across different cognitive tasks. Generally, the ToT effect proved to be consistently replicable in terms of a close replication, i.e., using samples and items drawn from identical populations. A negative ToT effect was found for a figural reasoning task, meaning faster answers tended to be correct on average. In line with the accounts on dual processing and cognitive load, the effect was moderated by item difficulty and person ability. For less able test-takers solving difficult items, slower answers had a higher probability of being correct in relative terms. This pattern was replicable for different samples of persons and items. By contrast, the generalizability of the ToT effect across different tasks was only moderate, suggesting a relevant role of task-dependent moderators and rendering it likely that the employed tasks tap different solution processes.

## Figures and Tables

**Table 1:** Descriptive statistics and correlations of cognitive performance measures.

		Accuracy		Response Time		Correlations			
	*N*	*M*(*SD*)	ω	*M*(*SD*)	ω	FR	NR	VR	SK
Figural Reasoning (FR)	2080	.64(.26)	.94	54.44(14.46)	.77	—	.16	.25	.24
Numerical Reasoning (NR)	2640	.53(.18)	.68	57.26(6.83)	.76	.33	—	.29	.30
Verbal Reasoning (VR)	2640	.63(.14)	.55	56.75(7.07)	.81	.28	.36	—	.27
Sciences Knowledge (SK)	2640	.49(.18)	.68	57.19(17.64)	.75	.31	.40	.24	—

Note: Response time in seconds. ω = McDonald’s omega. Uncorrected accuracy correlations are below the diagonal, uncorrected response time correlations are above the diagonal. All correlations are based on raw test scores and significant at *p* < .001.

**Table 2:** Descriptive statistics and correlations of the time on task effects.

			Model Parameters					Reliability	$b_{1p}$ Correlations			
	*N*	Items	$\beta_{1}$	$\sigma_{b1p}^{2}$	$\sigma_{b1i}^{2}$	*r* $(b_{0p},b_{1p})$	*r* $(b_{0i},b_{1i})$	$\rho_{b1p}$	FR	NR	VR	SK
FR	2640	28	−0.60	0.92	0.86	−.48	−.24	.41	—			
NR	2640	16	−0.23	0.36	0.76	−.50	−.77	.22	.16	—		
VR	2640	16	0.34	0.26	1.08	−.21	.05	.04	.08	.08	—	
SK	2640	20	−0.08	0.31	0.64	−.60	.03	.40	.16	.22	.09	—

Note: Model implied time on task effects for the figural reasoning task are based on ten separate models for the 10 subsamples. FR = Figural Reasoning, NR = Numerical Reasoning, VR = Verbal Reasoning, SK = Sciences Knowledge. $\rho_{b1p}$ = uncorrected split-half reliability of $b_{1p}$ estimates. All correlations are significant at *p* < .001.

## Data Availability

Due to data privacy restrictions, data cannot be shared.

## Supplementary Materials

The following supporting information can be downloaded at: https://www.mdpi.com/article/10.3390/jintelligence11050082/s1, Figure S1: Forest plot of time on task estimates for the figural reasoning task.
