# Supplementary material for: Testing Replicability and Generalizability of the Time on Task Effect
Source: J Intell. 2023 Apr 28;11(5):82. doi: 10.3390/jintelligence11050082 (PMC10219143; doi:10.3390/jintelligence11050082)
Supplement: Supplementary file 1 [file jintelligence-11-00082-s001.zip › jintelligence-2119036-supplementary.pdf]

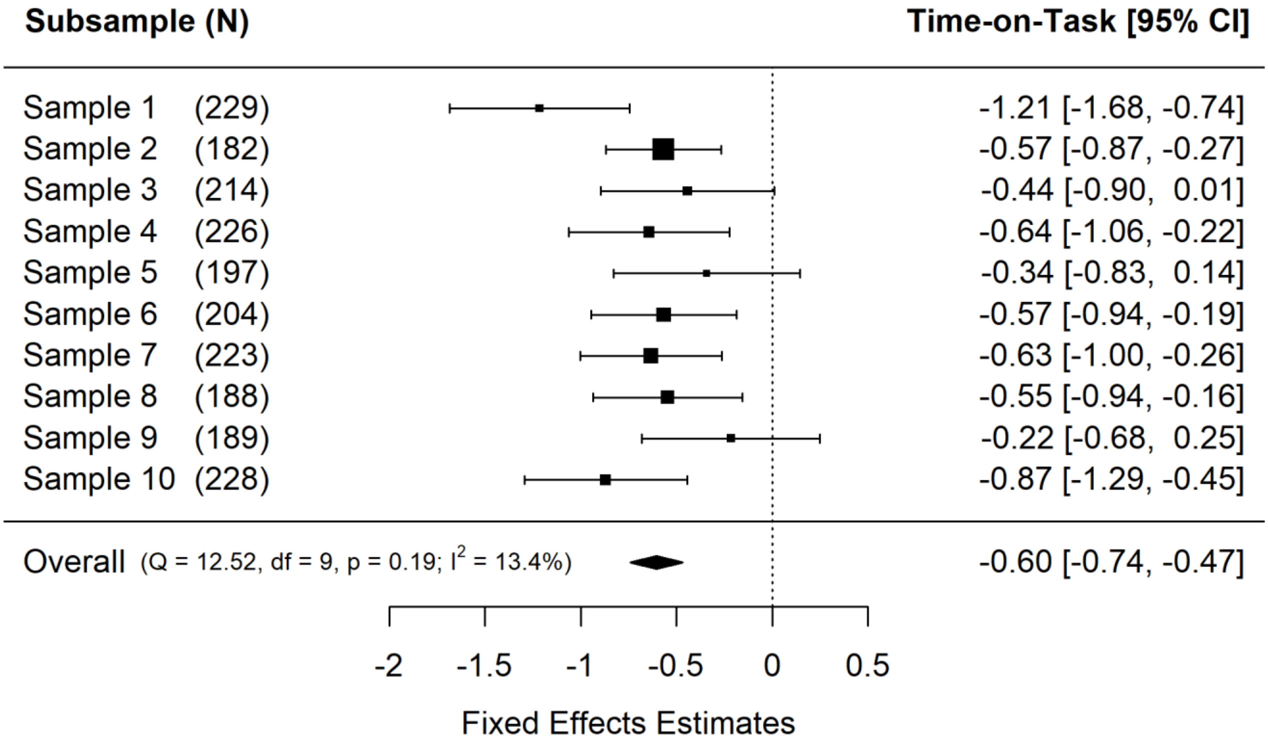

Figure S1. Forest plot of time on task estimates for the figural reasoning task. Random effects meta-analysis. Sizes of the 10 samples are given in parentheses. Bars represent 95% confidence interval.
